# Effect of PLA/PBAT Antibacterial Film on Storage Quality of Passion Fruit during the Shelf-Life

**DOI:** 10.3390/molecules24183378

**Published:** 2019-09-17

**Authors:** Rong Zhang, Wenting Lan, Jie Ding, Saeed Ahmed, Wen Qin, Li He, Yaowen Liu

**Affiliations:** 1 College of Food Science, Sichuan Agricultural University, Yaan 625014, China; zhangronglzy@163.com (R.Z.); 18227593253@163.com (W.L.); saeedahmed_mahar@yahoo.com (S.A.); qinwen@sicau.edu.cn (W.Q.); 2 College of Food Science and Technology, Sichuan Tourism University, Chengdu 610100, China; dingjiedream@163.com; 3 School of Materials Science and Engineering, Southwest Jiaotong University, Chengdu 610031, China; 4 California NanoSystems Institute, University of California, Los Angeles, CA 90095, USA

**Keywords:** passion fruit, PLA/PBAT antibacterial films, storage quality

## Abstract

In this experiment, we studied the effect of poly(lactic acid)/poly(butylene adipate-co-terephthalate) (PLA/PBAT) blend films on the efficiency of passion fruit preservation at 20 °C. The weight loss, shrinkage index, firmness, and total sugar of passion fruit packaged with PLA/PBAT films had no significant differences compared with PE films during 21 days (*p* > 0.05). PLA/PBAT films can more effectively reduce the rising of ethanol content and delay the total acid, ascorbic acid, and sensory evaluation. Compared with unpackaged (CK) and polyethylene (PE) films, PLA/PBAT films are more conducive to preserve the overall flavor of passion fruit during storage time, in agreement with sensory evaluation, tested by E-nose, E-tongue, and GC-MS, which also proved that it can effectively maintain the edible quality of passion fruit during storage time. We believe that our study makes a significant contribution to literature because it paves the way to the generalization and application of packaging films based on composite antibacterial polymers and facilitates the commercialization of fresh passion fruit as an important health food.

## 1. Introduction

Purple passion fruit (*Passiflora adults Sims*) is a perennial evergreen climbing vine, which carries ripe fruit between August the same year and March the next year [1]. The ripened fruit, called “fruit monosodium glutamate”, tends to have bright color, unique and rich aroma, whose juice can emit complex aroma of pomegranate, pineapple, strawberry, and other fruits [2]. In recent years, passion fruit, characterized by distinct flavor and ample nutrition is a standing and popular product that is targeted to the Chinese fresh fruit market. Motivated by vigorous market demand and backed up by the government policy, the passion fruit planting area has been strengthened year by year in China. Now, purple passion fruit production is an important source of income for small-holder farmers in the poverty-stricken districts of southern China province. However, high temperature in harvesting season makes passion fruit, as a typical kind of respiratory climactic fruit, highly susceptible to water loss, which easily leads to fruit shrivelling and causes a reduction in juice quality and physiological deterioration, to produce a peculiar smell during storage, therefore resulting in a short shelf-life [3]. In order to reduce the loss of postharvest, many researchers focused on cheaper and more effectual preservative methods.

Many studies have been focused on nutritional composition as well as medicinal potential of *Passiflora foetida L*. plant extracts and passionflower fruit juice processing [4]. The preservation techniques mainly related to calcium treatment, hot-water treatment, 1-Methylcyclopropene (1-MCP) treatment, and modified atmosphere packages. A. Baraza et al. suggested that commercial application of 1-MCP in passion fruit at turning stage (60–65 days after anthesis) was optimal to ensure efficacy, since they were still marketable on day 14 [5]. Jacqueline Barbosa Dutra et al. found that the combination of hot-water treatment at 47 °C (4 or 5 min) and application of the phosphite of K or Zn could significantly reduce the disease severity in fruits; the 1-MCP treatment reduced the anthracnose severity in passion fruit mainly at 200 nL L^−1^ · 24 h^−1^ [6]. Da Silva et al. studied that the application of CaCl_2_ by infiltration favored the maintenance of fruit consistency during the first 18 days of storage time [7]. Maniwara, P. et al. used modified atmosphere packaging (MAP) with an oxygen transmission rate of 12,000 cm^3^/m^2^∙day∙atm and showed the best result in maintaining fruit quality, gas composition, and extension of storage life (up to 51 days) [8]. The high potential of the atmosphere modification technique and the growing interest in large-scale retail trade in fresh berry fruit has drawn increased attention to the development of novel food packaging techniques, particularly those avoiding environmental pollution with plastic via the use of biodegradable bio-based polymers [9,10,11].

Covered passion fruit with polyethylene (PE) film packaging is widely used in China market, because the packaging enhances the shelf-life and quality of passion fruits [12,13]. However, it is difficult to degrade and easily leads to serious plastic pollution. The market urgently needs the products with the same fresh-keeping effect but environment-friendly packaging to replace PE fresh-keeping films. Lactic acid-based polymers are known as poly(lactic acid) (PLA), which has high mechanical properties, thermal plasticity, and fabric ability, and these monomers are getting more and more attention [14]. Some previous studies were performed to improve performance properties (such as impact strength, flexibility, stiffness, gas barrier properties, and thermal stability) of PLA, using addition of modifiers, blending, compatibilization, and physical treatments to enhance the quality of stored food [15]. In order to attend to the growing consumer demand for innovative ready-to-eat fresh, cut fruit packaging, PLA-based packages were widely investigated [16]. PLA/Ag/Vitamin E membrane actively reduced the polyphenol oxidase activity, which could have application in the food industry as a potential preservative packaging for fruit and juice [17]. The flexibility of PLA film would be increased significantly by blending a small amount of poly(butylene adipate-co-terephthalate) (PBAT), and it was convenient to retain the freshness of packaged green onion [18]. Although PLA/PBAT blend film is well known for exhibiting excellent physical properties, its use to preserve passion fruit has not been extensively reported. Present reports of using the electronic tongue and nose on the effect of different packaging films on the edible quality of passion fruit during the shelf-life are limited.

Therefore, this work aimed to study the effect of PLA/PBAT blend films on the efficiency of passion fruit preservation at 20 °C; nonpackaged and PE films was used as control groups. This study also aimed to characterize the volatile compounds responsible and taste profile for the flavor of each state of passion fruit ripeness, using electronic tongue and nose.

## 2. Results and Discussion

### 2.1. Weight Loss

**Figure 1:**
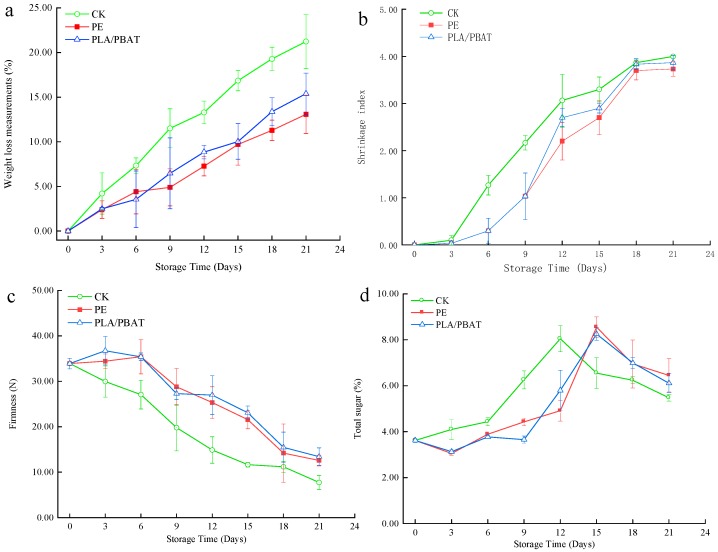
Effect of PLA/PBAT antibacterial packaging film on the (**a**) weight loss, (**b**) shrinkage index, (**c**) firmness, and (**d**) total sugar of passion fruit during storage times (t = 8). PLA: poly(lactic acid); PBAT: poly(butylene adipate-co-terephthalate); CK: unpackaged; PE: polyethylene.

Passion fruits are climacteric fruits which exhibit a short postharvest life due to rapid water loss and shriveling, which contributes to loss in marketability [5]. Weight loss was mainly ascribed to respiration and moisture evaporation through the passion fruit skin and resulted in fruit shrinkage and deterioration. The water-loss rate was determined by the water pressure gradient between fruit and the surrounding atmosphere and storage temperature [19]. Figure 1a shows the weight loss of passion fruits during 21 days room-temperature storage, revealing that all samples exhibited a continuous weight loss with time, with the greatest loss observed for the unpackaged (CK) sample. The weight loss of the CK sample after twelve days reached 11.52%, which was significantly higher than that of packaged samples (*p* < 0.05), since it was primarily caused by the migration of water from the fruit to the environment [20]. PLA/PBAT and PE packaging treatments can all effectively reduce the weight loss of passion fruits compared with the unpackaged sample. For packaged samples, the presence of packaging materials semipermeable to carbon dioxide, oxygen, and moisture decreased the extents of respiration, water loss, and oxidation, which was reflected in decreased weight loss [21]. Though PLA/PBAT films had higher oxygen permeability and water vapor permeability than PE, no significant difference in the weight loss was observed between the two packaged treatments. So, PLA/PBAT can effectively delay the descending rate of fruit weight loss compared with PE.

### 2.2. Shrinkage Index

Fruit shrinkage of peel is one of the important indicators to evaluate the storage quality of fruit, which can seriously affect the appearance quality of fruit, thus affecting their commercial value. Figure 1b shows there were no significant differences in these treatments at earlier storage (from 1 to 3 days). The shrinkage index of all samples trended towards an increase in the subsequent process of storage (from 6 to 21 days). During the storage period, the CK sample was observed with the greatest shrinkage index., which was significantly higher than those of packaged samples (*p* < 0.05). The shrinkage index of the CK sample after 12 days reached 3.07, thus the commodity value was less. The shrinkage of PE and PLA/PBAT treatments increased slowly during the first 9 days compared with CK, and rapidly exceeded 3.0 until the 18^th^ day. However, no significant difference in the shrinkage index was observed between the two packaged treatments (*p* > 0.05). There were great differences between the PLA/PBAT and PE groups at the 9th day (*p* < 0.05), but they had a similar tendency in the subsequent process of storage (from 12 to 21 days), when the two groups showed no significant difference (*p* > 0.05). Thus, for reducing the shrinkage index of passion fruit in storage, PPLA/PBAT and PE packaging films will have the same effect.

### 2.3. Firmness

Firmness had excellent correlation to storage quality of fruit and vegetables, and can take into consideration the process of ripening and senescence in fruit. Figure 1c shows the firmness of passion fruit during 21 day room temperature storage, revealing that all samples exhibited a continuous drop with time, with the greatest loss observed in the CK sample. Firmness values measured at earlier storage (from 1 to 6 days) were similar for all samples (*p* > 0.05) and began to be varied after nine days (*p* < 0.05; the firmness of samples significantly decreased after six (unpackaged) and nine (PLA/PBAT and PE) days of storage. After the 9th day of storage, firmness losses of 41.61%, 10.40%, and 19.51% were found in unpackaged, PE- and PLA/PBAT-packaged passion fruit, respectively (i.e., packaging helped to retain firmness). The PLA/PBAT group had no significant differences in comparison with the PE group during the latter stage of storage (from 12 to 21 days) (*p* > 0.05). Thus, PLA/PBAT can more effectively delay the descending rate of fruit firmness compared with PE.

### 2.4. Total Sugar

The quality and consumer perception of fruit is strongly affected by total sugar. Figure 1d presents the results of total sugar analysis during 21 d storage, revealing that total sugar of all groups increased first and then decreased, then the total sugar peaks of CK and packaging groups were reached at the 12th and 15th day, respectively. During this period, the increase rate of the CK group was significantly greater than that of other treatments. Starting from day 15, the sharpest increase of total sugar was observed for PLA/PABT- and PE-packaged samples (8.25% and 8.55%, respectively), but the difference between the two groups was not evident, while the total sugar in the CK group dropped to about 6.55% at the same time. After the peak of total sugar appeared, the PLA/PBAT group had no significant differences in comparison with the PE group during the latter stage of storage (from 15 to 21 days) (*p* > 0.05). Thus, PLA/ PBAT can also effectively reduce the senescence of passion fruits and delay the appearance of total sugar peak compared with PE.

### 2.5. Total Acid

**Figure 2:**
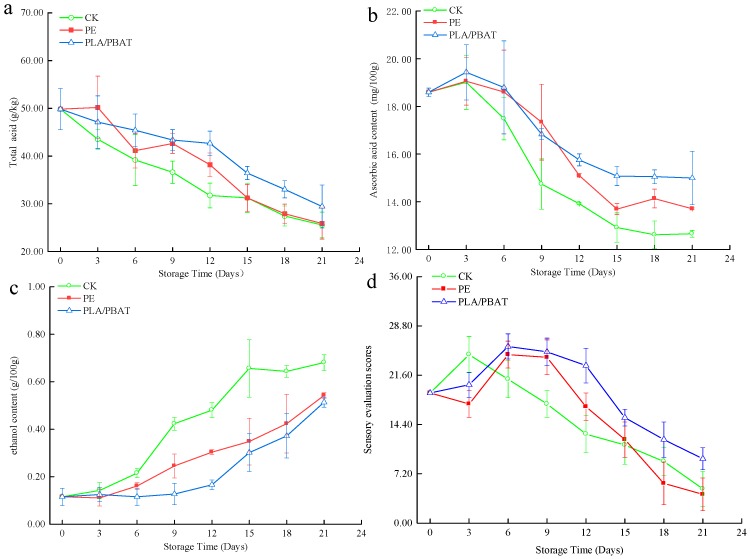
Effect of PLA/PBAT antibacterial packaging film on the (**a**) total acid, (**b**) ascorbic acid, (**c**) ethanol content, and (**d**) sensory evaluation of passion fruit during storage times (t = 8).

After soluble sugar, organic nonvolatile acids are the second most significant contributor to fruit flavor. Respiration, resulting in untreated acid consumption and fruit senescence, is thought to be one of the main reasons for total acid (TA) changes [22]. Figure 2a shows the effect of packaging on passion fruit total acid content, demonstrating that in all cases, TA decreased with time during storage, with the slowest decrease observed for fruit packaged in PLA/PBAT film. This result demonstrated that packaging materials can delay the utilization of organic acids and hence slow down the TA reduction, in agreement with our previous report [23]. In particular, the TA decrease (49.83 to 29.45 (1 g citric acid kg^-1^) over 21 d) observed for PLA/PBAT-packaged fruit was higher than that observed for PE-packaged samples, which was ascribed to the antibacterial effect of PLA/PBAT and its ability to slow down the deterioration of fruit during storage. Thus, packaging materials significantly (*p* < 0.05) reduced the rate of TA reduction. These were signs that PLA/PBAT can more effectively reduce the senescence of passion fruit and delay the total acid reduction than PE. This proves that PLA/PBAT can effectively maintain the edible quality of passion fruit during storage.

### 2.6. Ascorbic Acid Content

Figure 2b displays the ascorbic acid content of passion fruit in all treatment groups, which showed a downward trend. After storage, the ascorbic acid content of all samples decreased (*p* < 0.05), with the lowest value observed for the unpackaged sample (12.65 ± 0.14 (mg/100 g) at 21 d) and the highest value observed for the PLA/PBAT-packaged sample (15.00 ± 1.12 (mg/100 g) at 21 d). Ascorbic acid content measured at earlier storage (from 1 to 9 days) was similar for all packaged samples (*p* > 0.05) and began to get varied after nine days (*p* < 0.05), with that of the PE group significantly decreased compared with the PLA/PBAT group. Thus, PLA/PBAT can more effectively delay the ascorbic acid oxidizing than PE. Ascorbic acid of fruit was best preserved in the case of PLA/PBAT-packaged samples. In particular, the score decrease (25.8 ± 1.83 to 9.40 ± 1.60 over 21 d) observed for PLA/PBAT-packaged fruit was lower than that observed for PE-packaged samples, which was ascribed to high gas permeability of PLA/PBAT and its ability to slow down the ethanol production of fruit during storage.

### 2.7. Ethanlo Content

The high-density peel serves as a barrier that obstructs gas permeability into the fruit’s internal cavity. Anaerobic respiration produces acetaldehydes and ethanol as it ferments [8]. During the experiment, ethanol concentration in passion fruit markedly rose in the storage period in all groups (Figure 2c). Ethanol reached up to 0.42 g/100 g within 9 days under CK. The juice gathered from passion fruit packaged in PE reached the same amount during 18 days. Ethanol reached up to 0.42 g/100 g within 9 days under no packaging (CK). The fruit obtained from passion fruit packaged in PE reached the same amount in 18 days, with the lowest value observed for the PLA/PBAT sample (0.37 ± 0.09 (mg/100 g) at 18 d). Figure 2c provides the evidence that PLA/PBAT packaging delayed the rising of ethanol content in fruit for over 3 days when compared with the value obtained from PE packaging (12 day for PE and 15 day for PLA/PBAT).

### 2.8. Sensory Evaluation

Figure 2d presents the results of sensory evaluation analysis during 21 d storage, revealing that the score of all groups increased first and then decreased, then the sensory score peaks of CK and packaging groups were reached at 3 and 6 days, respectively. With PLA/PBAT and PE packaging, the flavor fell below the optimum level (score > 20) after 12 days and 9 days, respectively. Thus, packaging materials significantly (*p* < 0.05) reduced the rate of decline in sensory quality during the early and medium storage periods (from day 6 to day 12). The sudden increase in the flavor intensity over time in the early storage period may be due to the increase in the volatile compounds, particularly esters in fruits [24], while the epidermis of the fruit has not yet shrunk due to a large amount of water loss, maintaining a good color. This result demonstrated that, compared with PE, PLA/PBAT can reduce oxygen-free breathing of packaged fruit due to high gas permeability, and hence slow down the odor caused by ethanol production, in agreement with our previous report [19]. The antimicrobial effect of PLA/PBAT packaging film effectively delayed the process of fruit decay and deterioration caused by bacterial and fungal diseases, and the edible quality of the fruit was significantly better than that of other treatments in whole storage.

### 2.9. Intelligent Sensory Evaluation

**Figure 3:**
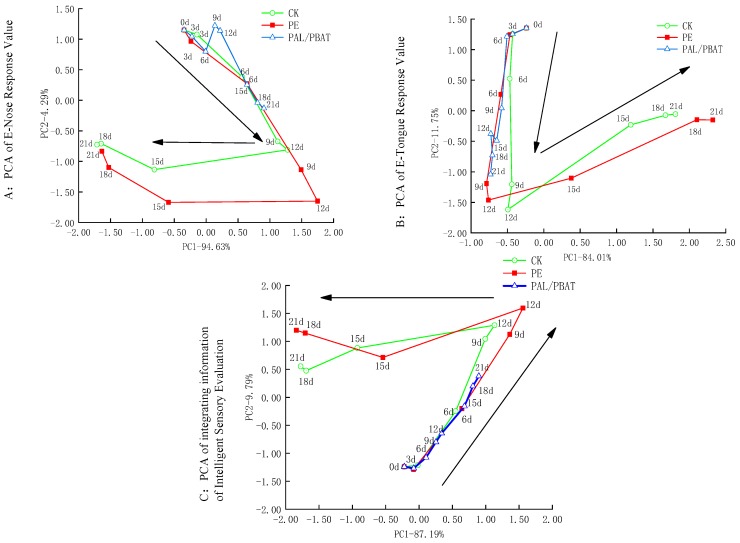
Effect of PLA/PBAT antibacterial packaging film on the (**a**) e-nose response signal, (**b**) e-tongue response signal, (**c**) fusion information of e-nose and e-tongue response of passion fruit during storage times (t = 8), based on principal component analysis (PCA). Note: All black arrows indicate the direction of displacement in PC1.

Figure 3a demonstrates that the variance contribution rates of PC1 and PC2 were 94.63% and 4.29% respectively, and the cumulative contribution rate was 98.92%, which contains most of the electronic nose feature information. The changes of electronic nose responses in unpackaged (CK) and PE-packaged groups could be divided into two stages: 0–12 d and 12–21 d, while the PC1 of electronic nose responses of CK and PE groups changed positively in the first stage, and negatively in the second stage. However, the PC1 of electronic nose responses of the PLA/PBAT group created a slight variation during the whole storage period, which was always in the first stage (0–12 d) sectioning of reference CK and PE. Thus, we can conclude that PAL/PBAT treatment had a smaller change in aroma profile than CK and PE treatment during 21 days, which had no significant difference between the aroma profile at the end of storage and at the medium period of storage (*p* > 0.05).

Figure 3b shows that the variance contribution rates of PC1 and PC2 were 84.01% and 11.75%, respectively, and the cumulative contribution rate was 95.76%, which contains most of the electronic tongue feature information. This was in line with the change of sensory evaluation and electronic nose response signal, indicating that the fruit flavor quality of CK and PE groups became worse suddenly at the 12^th^ day. The PC1 variation distance of CK and PE groups in the first stage (0–12 d) was 0.26 and 0.52 units, respectively, and that of the second stage (12–24 d) was 2.30 and 3.08 units, which is 9.03 and 5.92 times of that in the first stage. PC1 of PAL/PBAT showed a displacement in a positive direction during the whole storage period, which proved that taste profile of passion fruit in PAL/PBAT packaging film had smaller changes than that of CK and PE groups during the storage period of 21 days.

Figure 3c shows the results of principal component analysis of fused information features after homogenizing the response values of probes of e-nose and e-tongue. Variance contribution rates of PC1 and PC2 fused with information characteristics were 87.19% and 9.79%, respectively, and the cumulative contribution rate was 96.98% and over 85%, while PC1 was the main factor contributing to the change of intelligent sensory characteristics processed. In the first stage, PC1 of CK and PE were oriented positive shift from 0 to 12 days, and in the second stage, it changed negatively from 12 to 21 days. The PC1 of PAL/PBAT showed a displacement in a positive direction during the storage period, compared with CK and PE. In conclusion, PLA/PBAT film is more conducive to preserving the overall flavor of passion fruit during storage compared with CK and PE, in agreement with sensory evaluation.

### 2.10. Profile of Volatiles of the Passion Fruit

Table A1 shows that volatile and odoriferous compounds of the passion fruit in different packaging, corresponding to the 0^th^ day, 12^th^ day, and 21^st^ day by GC-MS, contain esters, alcohols, ketones, alkenes, aldehydes, and six other kinds of compounds. A total of 140 flavor components were identified this time. All treatment groups contained ethyl acetate, ethyl propionate, methyl butyrate, ethyl hexanoate, n-hexanol, and methyl heptanone during the whole storage. The dominant volatile substances of passion fruits during the storage period were esters, and the most important representative compounds were ethyl butanoate and ethyl acetate. Packaging materials have great influence on flavor substances of passion fruit during a room-temperature storage period. Ethyl acetate is a colorless clarifying liquid with aromatic odor [25,26]. Ethyl butanone has a clear and intense sweet fruit aroma, with pineapple, banana, and apple flavor, which spread easily but was not lasting, and with fat odor at high concentration [27]. The ethyl acetate concentration of PLA/PBAT-packaged and no-packaged (CK) markedly rose in the 12^th^ day (*p* < 0.05) and reached up to 31.84% and 10.49%. Meanwhile, the content of PE decreased from 24.54% in fresh fruit to 13.76%. The ethyl acetate concentration of PLA/PBAT-packaged and no-packaged (CK) markedly rose in the 12^th^ day (*p* < 0.05), reaching up to 31.84% and 10.49%. Meanwhile, the content of PE decreased from 24.54% in fresh fruit to 13.76%. Fresh passion fruit does not contain ethyl butanoate, which was gradually produced with fruit ripening and senescence. The ethyl butanoate concentration of PE- and PLA/PBAT-packaged markedly rose in the 12^th^ day (*p* < 0.05), reaching up to 20.09% and 43.96%,above CK (29.16%), but there was no significant difference at the end of storage time (21^st^ day) (*p* > 0.05).

Table A2a,b show that there were 39 flavor substances identified in the fresh passion fruit, containing six kinds of esters (14.41%), 17 kinds of terpenoids (64.39%), four ketones (3.55%), two kinds of aldehydes (0.38%), and 10 other classes (17.28%), especially for alcohols, who were detected at the 12^th^ or 21^st^ day. At the 12^th^ day, the ester content of each group increased significantly compared with 0 days, with the highest value observed for the PLA/PBAT sample, while the alcohol substance was significantly lower. At the end of storage time, the ester content of the PAL/PBAT group slightly increased, when compared with 12 days, while the alcohol substance dropped further. In conclusion, PLA/PBAT film is more conducive to preserving the volatile aroma components of passion fruit during storage compared with CK and PE groups, in agreement with sensory evaluation and electronic nose response signals.

## 3. Materials and Methods

### 3.1. Materials

Fresh, mature, purple passion fruit (*Passiflora edulis Sims,* aged 80 days after flowering) was harvested from Xinhongdao Lemon Agriculture Co., Ltd. located in Anyue, Sichuan province, China. The plantation site is at an altitude of 450 m above sea level, with an average temperature of 16.6~18.3 ℃. The fruit was selected for symmetrical spherical shape, weight ranging from 56 to 85 g, and purple color stage (color break) of 70–80 percent, transported to the laboratory in a cooled state, and used for packaging experiments on the same day. Defective fruit (i.e., those showing signs of decay and mechanical damage) were eliminated. Passion fruit of uniform size, color, and shape were consequently used in the above experiments.

In the antibacterial experiment, the packaged films with the same thickness (0.025 mm ± 0.003 mm) were selected with good sales and good reputation in Chinese market at present. PLA/PBAT antibacterial packaging films (food grade) were obtained from Kangrunjie Environmental Protection Technology Co., Ltd. (Jilin, China). PE packaging films (food grade) were obtained from Suzhou Jieda Chemical Plastics Co., Ltd. (Zhejiang, China).

### 3.2. Fruit Preparation and Packaging

Passion fruits were randomly divided into third groups of 70 unit. Two groups were wrapped fruits in PLA/ PBAT- and PE-packaged films, and the third group contained control (unpackaged) passion fruit. Passion fruit in all packaging conditions were sampled immediately after packaging, after 12 h, and then in 3 days intervals thereafter. Packaged and control samples were evaluated after 0, 3, 6, 9, 12, 15, 18, 21 d storage time. All samples were placed into plastic boxes and stored at 20 ± 1 °C and relative humidity (RH) of 70–75 %, a condition based on the common temperature of a display case with air-cooler in most Chinese supermarket. Three packaging samples from each packaging condition were tested, totaling 15 fruit per packaging type. Five fruits were used for chemical quality analysis, and 10 fruits for sensory evaluation.

### 3.3. Quality Evaluation of Storage

The quality of passion fruits was evaluated every two days, for a total of 21 d. Each data was collected from at least three separate experiments. Error bars were used to indicate the estimated error in a measurement or the uncertainty in a value.

#### 3.3.1. Weight Loss Measurements

Weight loss during storage was measured daily using an FA 2004W balance (±0.0001 g; Shanghai Jinghai Instrument Co., Ltd., Shanghai, China) and expressed as (W0 – Wt)/W0, where W0 and Wt are initial weight and tested-date weight, respectively.

#### 3.3.2. Shrinkage Index Measurements

The shrinkage index of passion fruit was characterized every day according to a four-point empirical scale (Table 1) [28].

#### 3.3.3. Total Acid and Total Sugar Measurements

Total acid was determined for 25 g passion fruit juice samples by titration with 0.1 mol/L NaOH solution and expressed as % citric acid [6].

The determination of total sugar was calculated for 20 g passion fruit juice by the method of phenol-vitriol, in which dextrose is the standard sample. The concentration was given in spectroscopy at 490 nm with ultraviolet spectrophotometer (UV-Blue Star A, Beijing Leibotaike Instruments Co., Ltd., Beijing, China). The regression equations of the standard curves based on the concentrations (C) versus the absorptive value (A) are:

*y* = 0.0069*x* + 0.0002 (R^2^ = 0.9992).

#### 3.3.4. Firmness Measurements

Firmness was measured at room temperature using a food texture analyzer (FTA) (type TMS-PRO, American TFC Company.), equipped with a 1 kg load cell. A single whole passion fruit was placed on a flat platform and penetrated with a 2 mm diameter flat-head stainless-steel cylindrical probe to a depth of 15 mm at a speed of 1 mm s^−1^, when deformation and minimum induction force were set to 50% and 0.375 N, respectively.

#### 3.3.5. Ascorbic Acid Measurements

Ascorbic acid was determined for 25 g passion fruit juice samples by high-performance liquid chromatography (U3000, Thermo Fisher Scientific, Waltham, MA, USA), in which L ascorbic acid was the universal sample. Ascorbic acid in the sample was drawn with 20 g/L metaphosphate solution by ultrasound for 5 min; a mixture of 1% acetonitrile and 99% of 0.01 mol/L potassium dehydration phosphate (pH 2.5) as the mobile phase. Reduction of L(+)-dehydroretinol acid in the sample by L-cysteine solution were separated on a C18 reverse-phase chromatography column and measured by ultraviolet detector (wavelength = 246 nm). The regression equations of the standard curves based on the concentrations (C) versus the absorptive value (A) are:

*y* = 0.1361*x* + 0.0325 (R^2^ = 0.9993)

#### 3.3.6. Ethanol Measurements

Ethanol was determined for 50 g passion fruit juice samples, based on the number of ISO 2148-1973, which is titled “Fruit and vegetable products—Determination of ethanol”.

#### 3.3.7. Sensory Evaluation

The evaluation was performed by 10 trained panels, consisting of five females and five males (age 20–34). All panelists had a professional background in Food Science and Engineering and were trained in evaluation passion fruit, rated from three aspects, as the overall flavor, the degree of pericarp in good condition, and pericarp color [29]. The quality description grade included three grades: extremely poor, medium, and very good, with scores of 1, 5, and 9, respectively, and the specific standards are in Table 2. The summation of three scores was the sensory evaluation total score of the fruit.

#### 3.3.8. E-Nose and E-Tongue Measurements

The juices were marked for 2 g passion fruit juice samples by E-nose (FOX 4000, Alpha MOS, France). This apparatus mainly consists of a sensor array of 18 Metal Oxide Semiconductor (MOS) sensors, a signal collection system, and a data processing software. The details of the sensor array and the process of sampling detection by E-nose have been described by previous reports [30]. For E-noise detection, three samples were prepared for each group.

To detect the liquid fingerprint of 10 g passion fruit juice samples, which were filtered with the filter paper and then diluted into 80 mL with deionized water, an E-tongue (α-Astree, α MOS Company, France) was applied. The E-tongue is mainly made up of an automatic sampler, a sensor array, and a signal processing software. The details of the E-tongue sensor array and the process of sampling detection by E-tongue were previous reported [31]. For E-tongue detection, three samples were prepared for each group.

#### 3.3.9. Aroma of Organic Passion Fruit Measurements

Aroma of organic passion fruit was determined for 2 g passion fruit juice samples by A SQ680 gas chromatograph–mass spectrometer from Perkin Elmer (USA), with an electron impact ionization source (70 eV), used in scan mode. The mass range was from 35 to 400 m/z. The Elite-5MS column (30 m length, 0.25 mm i.d., 0.25 mm film thickness) was maintained at 40 ℃ for 2 min, then programmed to rise to 60 ℃ at 2 °C/min, and held for 1 min. Then programmed to rise to 250 °C at 20 °C/min, and held for 2 min. Helium (99.9999% pure) was used at a flow rate of 1 mL/min, which has a split ratio of 5:1.

The volatile compounds of the passion fruit in different packaging films were identified by GC-MS with Elite-5MS columns, comparing the mass spectra obtained with those of pure standards with those found in the National Institute of Standards and Technology (NIST) library (vers. 1.7), the determination was carried out by combining with the artificial analytical mass spectrometry. The peak area normalization method was utilized to determine the relative content of volatile components.

### 3.4. Statistical Analysis

Multiple samples were tested, and the results were reported as mean ± standard deviation. The obtained values were subjected to analysis of variance, and the means were separated by Duncan’s multiple range test (Super ANOVA, Abacus Concepts, Inc., Berkeley, CA, USA), with *p* values of <0.05 considered significant. The results were treated with principle component analysis (PCA) by SPSS19.0 software.

## 4. Conclusions

In the Chinese market, passion fruit is mainly sold as fresh fruit after ripening, but its pericarp is easily dehydrated and shrinks, which leads to the decline of appearance value and flavor quality. Suitable postharvest packaging would help to spontaneously form a small atmospheric environment, and have a significant impact on shelf-life extension of Passion Fruit by prevented losses from spoilage spreading, water loss, and shrinkage. In Chinese market, the packaging materials widely used in fruits and vegetables are mainly PE-based packaging materials at present. Compared with the blank control, PAL/PBAT and PE films can effectively prolong the shelf life of passion fruit. During passion fruit preservation experiments, PLA/PBAT and PE packaging slowed down the process of fruit metabolism and delayed ripening, as indicated by the concomitant changes of weight loss, shrinkage index, firmness, total sugar, total acid, ascorbic acid content, ethanol content, sensory evaluation, and aroma components. Compared with PE films, PLA/PBAT films are more conducive to preserve the overall flavor of passion fruit during storage time, in agreement with sensory evaluation tested by E-nose, E-tongue, and GC-MS, which also proved that it can effectively maintain the edible quality of passion fruit during storage time. However, compared with PE, PAL/PBAT films did not perform well in maintaining fruit color, shrinkage, and water loss. So the relevant difficulties in technology are still expected to be studied and explored, such as the optimum thickness and storage temperature of PLA/PBAT films.

## Figures and Tables

**Table 1:** Four-point systems used to evaluate the degree of passion fruit shrinkage.

Degree (severity) of Shrinkage	Area of Infected Fruit Surface (%)
0	0
1	0–25
2	25–50
3	50–75
4	>75

The shrinkage index = $\frac{\left(d\times f\right)}{n}$, where d is the category of shrinkage intensity scored on fruit, f is shrinkage occurrence frequency, N is the total number of examined fruit (both healthy and shrinkage).

**Table 2:** The score criterion of sensory evaluation of passion fruit.

Evaluation Item	The Description of Passion Fruit Quality	Score
Overall flavor	Excellent: it has nice taste and sweet smell.	1
	Moderate: it frees from foreign smell, with lighter flavors.	5
	Bad: it tastes too vinegary, or has serious peculiar smell.	9
Degree of pericarp in good condition	Excellent: it has an excellent surface appearance.	1
	Moderate: the pericarp area is more than 30% wrinkled, pitted or rotten.	5
	Bad: the pericarp area is more than 50% wrinkled, pitted or rotten.	9
Pericarp color	Excellent: it has good color and high gloss.	1
	Moderate: it has some slight color variation.	5
	Bad: it has an uneven color distribution and poor gloss.	9

## Appendix A.

**Table A1:** Volatile and odoriferous compounds of the passion fruit in different packaged, corresponding to the 0^th^ day, 12^th^ day and 21^st^ day by GC-MS.

Sequence Number.	Classification.	Components.	Retention Time.	Match.	R. Match.	Chemical Abstracts Service Number.	Relative Contents						
							0^th^ d	12^th^ d			21^st^ d		
							Fresh Fruit	CK	PAL/PBAT	PE	CK	PAL/PBAT	PE
1	Esters	Ethyl acetate	2.255	891	907	141-78-6	12.66	24.54	31.84	10.49	13.72	10.82	13.76
2		n-Ethyl propanoate	3.422	781	901	105-37-3	0.08	0.1	0.19	0.08	0.02	0.04	0.02
3		Methyl butyrate	3.642	894	905	623-42-7	0.06	0.24	0.63	0.38	0.32	0.03	0.32
4		Prenyl acetate	11.016	730	752	1191-16-8	0.22	0.21	0.21	0.13			0.09
5		Ethyl hexanoate	15.686	809	849	123-66-0	0.38	0.61	3.02	13.07	2.68	14.5	13.21
6		Hexyl butyrate	28.739	868	891	2639-63-6	1.02	2.85		6.01	3.42	4.78	2.11
7		Methyl acetate	1.786	793	809	79-20-9		0.31	0.27	0.12	0.17	0.08	0.08
8		Butyl acetate	6.082	898	920	123-86-4		0.5	0.6	0.28	0.37	0.39	0.36
9		Acetic acid-2-pentyl ester	7.3	584	769	626-38-0				0.03			0.09
10		Isoamyl acetate	8.698	804	860	123-92-2			0.22	0.07	0.02	0.1	0.1
11		Ethyl 3-hydroxybutyrate	11.64	776	849	5405-41-4			0.06	0.12	0.24	0.33	0.14
12		Pentyl acetate	8.698	789	843	628-63-7				0.07			
13		4-penten-1-yl acetate	9.009	751	829	1576-85-8				0.05			
14		Pentyl 2-methylpropanoate	9.754	704	825	2445-72-9				0.06			
15		Heptyl formate	13.885	818	885	112-23-2			0.16	0.2	0.66	0.16	
16		Isobutyl butyrate	15.474	703	832	539-90-2				0.15			
17		n-Butyl butanoate	15.474	755	873	109-21-7		0.07	0.12	0.15	10.29	0.1	0.07
18		Trans-3-hexenyl acetate	16.038	923	921	3681-82-1				4.98	0.13	4.33	
19		Hexyl acetate	16.545	917	918	142-92-7		1.22	2.63	4.6	0.14	2.74	3.82
20		Heptan-2-yl acetate	18.353	868	895	5921-82-4				0.17	0.06	0.21	0.28
21		Benzyl acetate	26.454	850	907	140-11-4		0.23		0.29	0.23	0.22	0.19
22		Cis-3-Hexenyl Butyrate	28.101	857	906	16491-36-4				0.06	1.08		
23		Ethyl butanoate	5.429	906	911	105-54-4		20.09	43.96	29.16	34.91	34.67	38.84
24		Ethyl caprylate	29.077	896	903	106-32-1				0.41	0.38	0.58	0.39
25		Heptan-2-yl butanoate	30.093	864	873	39026-94-3				0.75	0.74	0.95	0.59
26		Ethyl crotonate	7.135	848	857	623-70-1					0.22	0.39	
27		3-methylbutan-2-yl acetate	7.3	646	797	5343-96-4						0.03	
28		3-Methyl-3-buten-1-yl acetate	8.606	635	825	5205/7/2			0.09	0.05		0.03	0.03
29		Propyl butyrate	6.511	664	665	105-66-8					1.71	0.04	
30		(3Z)-3-Hexen-1-yl acetate	16.038	923	925	3681-71-8		2.76			0.13	4.33	3.36
31		Elaidic acid ethyl ester	21.784	453	493	6114-18-7							0.14
32		Ethyl hex-3-enoate	14.828	669	739	2396-83-0						0.05	
33		Hex-2-enoic acid ethyl ester	18.551	822	865	1552-67-6					0.78	0.09	0.15
34		Ethyl 6-heptenoate	21.839	585	783	25118-23-4						0.08	
35		(4-methyl-1-propan-2-ylcyclohex-3-en-1-yl) acetate	27.507	766	799	4821/4/9						0.59	0.08
36		Butanoic acid,(3E)-3-hexen-1-yl ester	28.064	920	940	53398-84-8						1.62	
37		Pentyl formate	4.603	585	821	638-49-3							0.03
38		Isobutyl acetate	547	717	804	110-19-0							0.02
39		Ethyl 3-hydroxyhexanoate	11.493	634	655	2305-25-1					0.02		
40		Ethyl 3-hydroxy-4-methylpentanoate	24.073	786	830	40309-42-0					0.02		
41		Ethyl pivalate	11.493	864	920	3938-95-2					0.47		
42		Vinyl butyrate	19.931	460	722	123-20-6					0.06		
43		Ethyl isobutyrate	22.763	685	771	97-62-1					0.07		
44		(Z)-3-Hexenyl isobutyrate	27.995	558	735	41519-23-7							0.02
45		Propyl acetate	3.451	700		109-60-4		0.07					
46	Terpenoids	Ethanol	1.628	936	938	64-17-5	2.09	1.72		0.67	1.68	2.03	1.61
47		1-Pentanol	4.662	888	895	71-41-0	1.64	0.32	0.12		0.12	0.14	
48		Leaf alcohol	7.703	930	943	928-96-1	1.14	2.59	3.31	1.4	5.85	1.78	0.97
49		1-Hexanol	8.338	867	869	111-27-3	5.95	5.72	8.13	4.84	0.12	4.43	3.65
50		Benzyl alcohol	17.722	745	824	100-51-6	1.24	0.36			0.15	0.16	0.06
51		Linalool	22.349	897	903	78-70-6	27.58	14.65		6.33	7.88		4.61
52		(-)-Terpinen-4-ol	27.584	730	744	20126-76-5	0.38						0.5
53		Alpha-Terpineol	28.615	881	917	98-55-5	3.59	2.23		1.26	1.55	0.7	0.79
54		2-methylbut-2-en-1-ol	4.871	490	725	4675-87-0				0.02			
55		Trans-3-Hexen-1-ol	7.582	768	818	928-97-2		2.67		1.4	0.04	1.84	
56		2-Heptanol	9.905	833	869	543-49-7				1.08	0.08	1.7	2.21
57		Sulcatol	15.272	753	818	1569-60-4			0.08	0.08	0.19	0.12	0.06
58		2-ethyl-1-hexanol	17.576	654	777	104-76-7				0.11	0.21		
59		Cis-oct-3-en-1-ol	18.867	637	729	18185-81-4				0.47			0.04
60		5-Methyl-2-hexanol	9.791	815	842	627-59-8						0.07	
61		2-Nonanol	22.459	741	810	628-99-9					0.39	0.12	
62		(Z)-1-hydroxy-1,3-butadiene	2.556	664	767	70415-58-6							0.09
63		2-[(2R,5S)-5-Methyl-5-vinyltetrahydro-2-furanyl]-2-propanol	20.213	612	739	5989-33-3	0.32	0.32	0.1	0.06			0.05
64		3-Methyl-2-buten-1-ol	4.871	489	696	556-82-1		0.15					
65		4-terpineol	27.408	762	769	562-74-3		0.03			0.64		
66		Cineole	17.268	510	667	470-82-6			0.23				0.1
67		Cis-3-octen-1-ol	18.643	713	871	20125-84-2			0.12		0.06		0.26
68		1-Chloro-1-propene	1.841	750	789	590-21-6	0.54						
69		1,5-Hexadiene	2.043	773	854	592-42-7	0.06						
70		α-phellandrene	11.134	639	755	99-83-2	0.18						
71		α-Pinene	11.478	873	897	80-56-8	0.68						0.14
72		3-Carene	11.478	870	901	13466-78-9	0.68				0.29		0.68
73		(+)-Limonene	17.407	900	903	5989-27-5	17.95						
74		Terpinolene	19.377	723	846	586-62-9	0.25		0.16	0.16	0.06	0.09	0.1
75		1,3,5,5-TetraMethyl-1,3-cyclohexadiene	24.282	562	743	4724-89-4	0.14						
76		β-pinene	14.997	567	732	127-91-3			0.03	0.81		0.62	
77		(-)-β-Pinene	14.876	561	722	18172-67-3					0.19	0.62	0.43
78		(Z)-β-ocimene	18.06	908	911	13877-91-3			1.24	1.07		0.6	
79		1-methylidene-4-prop-1-en-2-ylcyclohexane	30.658	773	840	499-97-8					0.2		
80		Cyclooctene oxide	7.582	765		286-62-4				0.2			
81		(Z)-β-ocimene	18.06	740	843	3338-55-4				0.24			
82		γ-Terpinene	19.348	740	822	99-85-4				0.1			
83		(4E,6Z)-2,6-dimethylocta-2,4,6-triene	24.26	751	845	7216-56-0				0.08			
84		α-Terpinene	20.969	791	844	99-86-5		0.19					
85	Ketones	3-methyl-2-hexanone	2.131	736		2550-21-2	2.1						
86		Cyclopentanone	5.271	533	793	120-92-3	0.55	0.46	0.11				
87		6-Methylhept-5-en-2-one	14.718	903	922	110-93-0	0.8	0.4	0.35	0.36	0.05	0.76	0.21
88		Ethyl (2Z)-but-2-enoate	7.248	841	875	6776-19-8				0.31			0.61
89		Cyclopentenone	6.742	580		930-30-3	0.1						
90		Hept-6-ene-2,5-dione	3.029	614	744	70353-50-3				0.06			
91		2-Nonenone-4	3.983	652	786	32064-72-5				0.03			
92		2-butan-2-ylcyclopentan-1-one	5.154	603	733	6376-92-7				0.03			
93		2-Heptanone	9.152	735	834	110-43-0				0.17	0.02	0.31	0.37
94		4-penten-2-one	2.89	583		13891-87-7		0.01					
95		2-Nonanone	21.615	599	832	821-55-6					0.02	0.15	0.09
96		3-Penten-2-one	3.91	650	860	625-33-2					0.05		
97		Pentan-2-one	2.982	597	688	107-87-9							0.09
98		l-menthone	25.782	424		14073-97-3							0.01
99	Aldehyde	(E)-2-Pentenal	4.343	760	814	1576-87-0	0.29	0.1		0.05		0.07	
100		3-Methyl-2-butenal	4.343	820	914	107-86-8	0.09				0.06		0.02
101		.β.-Methyl acrolein	2.556	830	880	4170-30-3						0.06	
102		Valeraldehyde	3.206	876	898	110-62-3		0.05	0.35			0.1	
103		Decanal	29.609	789	867	112-31-2		0.47			0.08		
104		Benzaldehyde	13.056	863	883	100-52-7		2.88	0.66	0.34	0.17	0.21	0.13
105		(E,E)-2,4-Heptadienal	16.328	722	882	4313/3/5		0.18			1.88	0.09	
106		Nonanal	22.679	780	829	124-19-6		1.97		0.92	0.33	0.25	0.53
107		Acetaldehyde	1.54	741	888	75-07-0				0.06	0.15		0.03
108		Methylpropenal	2.611	813	896	78-85-3				0.02			
109		(Z)-2-heptenal	12.931	806	921	57266-86-1				0.07			
110		Octanal	15.859	859	871	124-13-0		0.44	0.28	0.2			
111		(2E)-hexenal	7.597	697	814	6728-26-3		0.07			2.29		
112		Heptanal	9.886	865	894	111-71-7		0.61					
113		Tiglic Aldehyde	3.826	524	887	497-03-0			0.06				
114	Others	Ammonium carbamate	1.324	978	975	1111-78-0	0.34	0.44					0.12
115		Vinyl fluoride	1.485	735	830	1975/2/5	0.66						
116		2,3-dihydrofuran	2.615	724	879	1191-99-7	0.12	0.04					
117		Hexamethylcyclotrisiloxane	6.115	910	919	541-05-9	0.74	0.44	0.35	0.06	0.5	0.37	0.1
118		Ether	8.239	722	892	100-41-4	0.08						
119		o-Cymene	17.102	899	922	527-84-4	0.59	0.22					
120		Diallyl disulfide	20.65	483	695	2179-57-9	0.16						
121		(±)-Camphor	25.144	557	865	464-48-2	0.25						
122		Decamethylcyclopentasiloxane	25.504	925	916	541-02-6	14.27	5.84		4.5	0.03	0.27	1.13
123		Naphthalene	27.503	622	847	91-20-3	0.07						
124		(S)-(−)-Propylene oxide	1.705	854	899	16088-62-3		0.06		0.02	0.38		
125		2-propylfuran	16.299	717	812	4229-91-8		0.18	0.09	0.06			0.02
126		4-Heptenoic acid, ethyl ester, (E)	21.901	525	810	54340-70-4				0.07			
127		4-Methyl-2-(2-methylprop-1-en-1-yl)tetrahydro-2H-pyran	22.947	582	795	16409-43-1				0.3			0.12
128		2-Ethenyl-1,1-dimethyl-3-methylenecyclohexane	223.288	604	786	95452-08-7				0.1	0.34	0.07	0.08
129		3-Methylpyridazine	14.098	759	847	1632-76-4			0.04				
130		M-cymene	16.816	814	852	535-77-3			0.18				
131		Oxirane	1.448	876	912	75-21-8		0.17					
132		p-Xylene	8.235	674	808	106-42-3		0.04					
133		Phenol	14.384	771	919	108-95-2		0.14					0.04
134		Carbon dioxide	1.309	975	978	124-38-9							0.03
135		1,1-didodecoxyhexadecane	19.318	556	588	56554-64-4							0.05
136		Camphor	25.008	551	772	76-22-2							0.06
137		Methylhydrazine	1.478	720	901	60-34-4					0.89		1.51

Table A2 Effect of PLA/PBAT antibacterial packaging film on the VOCs composition of passion fruit during storage times (t = 3); (**a**) Fresh fruit without packaged. (**b**) PLA/PBAT and PE groups.

**Table:** (**a**)

Classification	Fresh Fruit					
	0^th^ Day		12^th^ Day		21^st^ Day	
	Sorts	Relative Contents	Sorts	Relative Contents	Sorts	Relative Contents
Esters	6	14.41	26	71.91	28	82.28
Terpenoids	17	64.39	19	20.37	15	15.02
Ketones	4	3.55	6	0.95	3	1.21
Aldehyde	2	0.38	7	1.66	6	0.78
Others	10	17.28	7	5.11	3	0.71

**Table:** (**b**)

Classification	PAL/PBAT				PE			
	12^th^ Day		21^st^ Day		12^th^ Day		21^st^ Day	
	Sorts	Relative Contents	Sorts	Relative Contents	Sorts	Relative Contents	Sorts	Relative Contents
Esters	14	84.00	28	73.07	14	53.80	26	78.29
Terpenoids	10	13.53	18	19.69	12	30.97	18	16.34
Ketones	2	0.46	4	0.14	3	0.87	6	1.39
Aldehyde	4	1.35	7	4.97	9	6.78	4	0.71
Others	4	0.67	5	2.13	10	7.59	11	3.26
